# Management of patients hospitalized for SARS-CoV-2 infection: A real-world economic evaluation from the hospital perspective

**DOI:** 10.1093/ajhp/zxag038

**Published:** 2026-02-11

**Authors:** Andre C Kalil, Mohsen Yaghoubi, Neera Ahuja, Ananth Kadambi, Thomas Oppelt, Christina G Rivera, Daniel R Kuritzkes

**Affiliations:** Division of Infectious Diseases, University of Nebraska Medical Center, Omaha, NE, USA; Evidence and Access, Certara, Radnor, PA, USA; Department of Internal Medicine, Stanford University School of Medicine, Stanford, CA, USA; Evidence and Access, Certara, Radnor, PA, USA; Medical Affairs, Gilead Sciences, Foster City, CA, USA; Department of Pharmacy, Mayo Clinic, Rochester, MN, USA; Division of Infectious Diseases, Brigham and Women’s Hospital, Harvard Medical School, Boston, MA, USA

**Keywords:** costs, COVID-19, remdesivir, SARS-CoV-2

## Abstract

**Purpose:**

SARS-CoV-2 infection continues to impact global health, particularly among high-risk vulnerable individuals. With the loss of federal funding for SARS-CoV-2 management, hospitals will need to budget appropriately for antivirals like remdesivir, which has demonstrated effectiveness in reducing mortality. We evaluated the economic impact realized by hospitals for patients hospitalized for SARS-CoV-2 infection and initiated on remdesivir therapy.

**Methods:**

We conducted a retrospective analysis using data compiled in the Premier Healthcare Database from January 2023 to February 2024. Propensity score matching was used to compare remdesivir-treated (RDV) and untreated (No RDV) groups. The mortality rate, hazard ratios associated with remdesivir use, and hospitalization costs were assessed overall and among the elderly (age ≥65 years). We also conducted a cost-effectiveness analysis to assess the economic value of remdesivir treatment.

**Results:**

Among 25,498 hospitalized patients, the mortality rate in the RDV group was lower than in the No RDV group (6.2% versus 8.1%), with a greater reduction in the elderly (6.9% versus 9.0%). Remdesivir significantly reduced mortality risk by approximately 25% overall and among the elderly. Each life saved was realized at a minimal increase in average hospitalization costs ($18,329 in the RDV group versus $14,845 in the No RDV group). Remdesivir was a cost-effective treatment option at a willingness-to-pay threshold of $25,000 overall and among the elderly.

**Conclusions:**

Our evaluation provides contemporaneous evidence of benefits and costs associated with management of individuals hospitalized for SARS-CoV-2 infection. Initiation of remdesivir was associated with minimal incremental hospitalization costs for lives saved as compared to not initiating remdesivir. Hospital pharmacy leadership can utilize this real-world evidence to appropriately budget for remdesivir treatment.

Key Points

- In this real-world study of over 25,000 hospitalized patients with COVID-19, remdesivir use was associated with a 1.9% absolute reduction in hospital mortality in the overall population and 2.1% reduction among the elderly.
- Despite slightly higher costs, remdesivir was cost-effective, supporting its use in high-risk patients under current endemic conditions.

Despite the transition of coronavirus disease 2019 (COVID-19) from the pandemic into an endemic phase, severe acute respiratory syndrome coronavirus 2 (SARS-CoV-2) infection continues to pose a substantial global health burden, with significant morbidity and mortality, particularly among high-risk groups such as older adults and those with underlying health conditions.^1–3^ COVID-19 remains a leading cause of death, ranking as the 10th leading cause in the United States in 2024, contributing to over 180,000 deaths.^4^ The ongoing impact of SARS-CoV-2 infection on healthcare systems is multifaceted, including persistent strain on hospital resources, rising healthcare costs, and continued economic loss due to extended illness and premature mortality.^5^ As of February 20, 2025, the overall rate of SARS-CoV-2–associated hospitalizations during the 2024-2025 respiratory virus season was 50 per 100,000,^6^ surpassing rates for other infection-related illnesses.^7^ Additionally, patients hospitalized for SARS-CoV-2 infection exhibited more than a 1.3-fold increased risk of mortality compared to patients hospitalized for influenza.^8^

Remdesivir has been the cornerstone antiviral treatment for patients hospitalized for SARS-CoV-2 infection since its emergency use authorization in May 2020 and subsequent full approval by the US Food and Drug Administration (FDA) in October 2020.^9,10^ As the first FDA-approved antiviral for COVID-19, remdesivir played a pivotal role in mitigating the severity of disease by reducing hospitalization duration^11^ and lowering mortality rates across different SARS-CoV-2 variants.^1^ Moreover, real-world effectiveness studies have confirmed the initial evidence from randomized controlled trials (RCTs), highlighting remdesivir’s consistent impact on reducing hospitalization duration and improving patient outcomes.^12,13^ A recent systematic literature review synthesized the totality of evidence on remdesivir clinical effectiveness, confirming its impact in hospitalized patients with COVID-19.^14^ Currently, remdesivir remains a key component of antiviral stewardship strategies and continues to be included in hospital treatment protocols.

During the 2023-2024 period covered by our study, major US and international clinical guidelines supported the use of remdesivir for hospitalized patients with COVID-19, particularly those requiring supplemental oxygen. For example, the National Institutes of Health (NIH) COVID-19 treatment guidelines recommended initiating remdesivir as soon as possible for hospitalized patients with moderate to severe disease who require oxygen, including low-flow or high-flow oxygen or noninvasive ventilation.^15^ Similarly, the Infectious Diseases Society of America (IDSA) guidelines aligned on remdesivir use in hospitalized patients with oxygen requirements and without contraindications.^16^ These recommendations reflect the prevailing standard of care during the study period and provide important contextual support for our findings on remdesivir’s clinical benefit and economic value in real-world hospital settings.

Since the clinical evidence supporting remdesivir initiation for SARS CoV-2 infection has been primarily informed by studies from the COVID-19 pandemic era and early endemic eras, there is an ongoing need to incorporate the most up-to-date learnings into current clinical practice to ensure remdesivir’s optimal use for management of SARS-CoV-2 infection. In addition, the financial landscape has shifted from an environment with substantial public health emergency funding to standard hospital budgeting of pharmaceuticals. During the pandemic, remdesivir was supported by government programs, ensuring widespread immediate access; however, as these emergency measures phase out, hospitals are integrating remdesivir into routine financial planning like all other pharmaceuticals in their settings.

Moreover, evaluations by health technology assessment (HTA) agencies, including the Institute for Clinical and Economic Review in the United States (US-ICER) and the National Institute for Health and Care Excellence (NICE) in the United Kingdom, suggested that remdesivir’s clinical benefits, combined with its demonstrated economic value, make it a key component of high-quality and efficient hospital-based SARS-CoV-2 infection management. However, their findings were tempered by the limited evidence available to quantify remdesivir’s survival benefit at the time.^17,18^ Our aim was to build on prior HTA research and evaluate the clinical and economic consequences of remdesivir treatment for patients hospitalized due to SARS-CoV-2 infection, focusing on monetization of lives saved based on routine clinical practice to enable hospitals to characterize the benefits of remdesivir realized for the costs incurred and guide their financial planning.

## Methods 

We evaluated both the clinical effectiveness in terms of benefits realized (from routine clinical practice) in patients hospitalized for SARS-CoV-2 infection, as well as the economic impact to hospitals for the outcomes observed, from the hospital perspective in the United States. Effectiveness was assessed by determining remdesivir’s impact on in-hospital mortality. Economic outcomes were evaluated by computing overall hospitalization-related direct costs to the individual hospital.

In addition, we conducted a cost-effectiveness analysis and a supplementary cost-utility analysis (using quality-adjusted life-years [QALYs]) to estimate the economic impact of remdesivir treatment.

## Data source

Data for this study were sourced from the Premier Healthcare Database (PHD), a comprehensive repository aggregating deidentified hospital-level data from over 1,000 US hospitals representing approximately 25% of US hospitalizations. The PHD includes patient demographics, disease state, diagnosis at admission and discharge, billed services, and other relevant patient-level data, enabling evaluation of treatment outcomes and the calculation of overall associated costs.^19^ Fewer than 2% of records in the remdesivir-treated group (the RDV group) had missing or misclassified treatment cost information, including instances where remdesivir costs were recorded as zero despite treatment; these records were excluded from the RDV group. This approach minimized the impact of incomplete or inconsistent financial records arising because of the federal government providing free remdesivir to hospitals.

We conducted a retrospective analysis of hospitalized patients with a primary diagnosis of COVID-19 (International Classification of Diseases, 10th Revision, Clinical Modification [ICD-10-CM] code U07.1) admitted from January 2023 to February 2024 during the most recent Omicron era. The use of ICD-10-CM code U07.1 to identify COVID-19 hospitalizations in the Premier (PINC AI) Healthcare Database has been previously validated and shown to have high reliability, with a positive predictive value of 93.8% in inpatient settings.^20^ On this basis, no additional COVID-19 ICD-10 codes were required for case identification in this analysis. Patients either received remdesivir (the RDV group) or did not receive remdesivir (the No RDV group) upon admission during the baseline period (ie, the first 2 days of hospitalization). Cost and mortality data were derived from hospitalization records covering the period from admission to discharge. Exclusion criteria included individuals who were pregnant, had incomplete data, were discharged or died within the first 2 days, were transferred from hospice care or another hospital, or were admitted for an elective procedure. Patients who had been initiated on COVID-19 treatments at baseline, such as baricitinib, tocilizumab (even if in accordance with guidelines), or oral antivirals, were also excluded. In addition to the overall population, we also evaluated the elderly subgroup (age 65 years or older). These exclusions affected approximately 18% of the initial patient records, resulting in a clinically homogenous cohort of 25,498 patients suitable for evaluating remdesivir’s independent effect on in-hospital mortality and costs. This approach ensures internal validity while maintaining representativeness of routine inpatient practice across US hospitals.

### Statistical analysis

We used a propensity score (PS) matching approach to ensure the RDV and No RDV groups were comparable, to minimize confounding by indication, and to account for differences in SARS-CoV-2 management practices between hospitals.^9^ Propensity scores were estimated using multivariable logistic regression, incorporating a comprehensive set of baseline covariates such as age, sex, race, insurance type, comorbidity burden, and indicators of disease severity upon admission.

Overall mortality in the RDV and No RDV groups was determined by calculating the proportion of patients in each group who died during their hospital stay. To estimate the risk of mortality associated with remdesivir treatment, we also calculated the hazard ratio (HR) using a Cox proportional hazards model. Hospitalization costs were based on the total recorded costs in the PHD, encompassing all billed services, procedures, medications, and other related care expenses incurred during the hospitalization.

Economic outcomes were assessed by comparing total hospitalization costs between the RDV and No RDV groups. We also calculated the numbers needed to treat (NNT) to avoid one death in the overall population and in the elderly. In this analysis, NNT was derived from the absolute risk reduction in overall in-hospital mortality from admission to discharge and does not reflect 14-day or 28-day mortality estimates. To provide supporting evidence, we furthermore conducted a cost-effectiveness analysis. Age-specific life-years lost (LYLs) were estimated by applying US life expectancy estimates from the 2019 US Life Table to the mortality rate observed in each group.^21^ QALYs lost were calculated by multiplying LYLs by a general population utility weight reflecting average health-related quality of life in the absence of COVID-19. Utility values were age specific and derived from published population norms to account for differences in baseline quality of life across age groups.^22^ The difference in QALYs lost and LYLs between the RDV and No RDV groups was used to estimate QALYs gained and life-years gained (LYGs) with remdesivir treatment. We further computed a cost-effectiveness ratio as the ratio of the difference in mean costs to the difference in QALYs lost.

This study was reviewed by Advarra institutional review board (protocol #Pro00091215) and was determined to be exempt from oversight in accordance with the Department of Health and Human Services regulations found at 45 CFR 46.104(d)(4).

## Results

Our evaluation included 25,498 patients hospitalized for SARS-CoV-2 infection equally divided into matched RDV (n = 12,749) and No RDV (n = 12,749) groups. Patient demographics are summarized in Table 1. A diverse geographic sample of US hospitals was represented. Both community hospitals and major medical centers across these regions are included, as reflected by bed sizes ranging from less than 100 to greater than 500, although nearly 60% of medical centers had 200 or more beds. Elderly individuals accounted for most of the population (∼84%), as reflected by a high percentage of Medicare beneficiaries (∼83%) compared to commercial insurance and Medicaid (collectively ∼13%).

**Table 1.** Baseline Demographics and Hospital Characteristics of Adults Hospitalized for SARS-CoV-2 infection – January 2023 to February 2024^a^

	No RDV (n = 12,749)	RDV (n = 12,749)
**Age group, years**		
18-49	441 (3.5)	441 (3.5)
50-64	1,591 (12.5)	1,591 (12.5)
65+	10,717 (84.1)	10,717 (84.1)
**Female sex**	6,806 (53.4)	6,773 (53.1)
**Race**		
White	10,027 (78.6)	10,060 (78.9)
Black	1,649 (12.9)	1,604 (12.6)
Asian	310 (2.4)	295 (2.3)
Other	763 (6.0)	790 (6.2)
**Ethnicity**		
Hispanic	926 (7.3)	896 (7.0)
Non-Hispanic	1,1116 (87.2)	1,1148 (87.4)
Unknown	707 (5.5)	705 (5.5)
**Primary payor**		
Commercial	997 (7.8)	1,016 (8.0)
Medicare	10,653 (83.6)	10,563 (82.9)
Medicaid	651 (5.1)	664 (5.2)
Other	448 (3.5)	506 (4.0)
**Hospital size, No. of beds**		
<100	1,029 (8.1)	1,036 (8.1)
100-199	2,191 (17.2)	2,184 (17.1)
200-299	2,582 (20.3)	2,537 (19.9)
300-399	2,322 (18.2)	2,306 (18.1)
400-499	1,380 (10.8)	1,441 (11.3)
**≥**500	3,245 (25.5)	3,245 (25.5)
**Hospital location**		
Urban	11,221 (88.0)	11,226 (88.1)
Rural	1,528 (12.0)	1,523 (11.9)
**Teaching hospital**	4,829 (37.9)	4,910 (38.5)
**Region**		
Midwest	2,754 (21.6)	2,705 (21.2)
Northeast	1,585 (12.4)	1,696 (13.3)
South	7,030 (55.1)	7,005 (54.9)
West	1,380 (10.8)	1,343 (10.5)
**Comorbid conditions**		
Obesity	3,011 (23.6)	3,028 (23.8)
COPD	5,032 (39.5)	5,067 (39.7)
Cardiovascular disease	11,730 (92.0)	11,755 (92.2)
Diabetes	4,949 (38.8)	4,928 (38.7)
Renal disease	4,360 (34.2)	4,357 (34.2)
Cancer	978 (7.7)	977 (7.7)
Immunocompromised condition	2,349 (18.4)	2,336 (18.3)
**Hospital ward upon admission**		
General ward	10,890 (85.4)	10,881 (85.3)
ICU/step-down unit	1,859 (14.6)	1,868 (14.7)
**Admission diagnosis**		
Sepsis	62 (0.5)	63 (0.5)
Pneumonia	842 (6.6)	892 (7.0)
**Other treatments at baseline**		
Anticoagulants	8,977 (70.4)	8,951 (70.2)
Convalescent plasma	0	0
Corticosteroids	8,847 (69.4)	8,892 (69.7)
Baricitinib	187 (1.5)	181 (1.4)
Tocilizumab	93 (0.7)	83 (0.7)
Oral antivirals	70 (0.5)	70 (0.5)
**Duration of remdesivir use, median (IQR), days**	NA	5 (4, 6)

Abbreviations: COPD, chronic obstructive pulmonary disease; ICU, intensive unit care; IQR, interquartile range; NA, not applicable; No RDV, remdesivir-untreated patients; RDV, remdesivir-treated patients; SARS-CoV-2, severe acute respiratory syndrome coronavirus 2.

^a^Data are presented as No. (%).

**Figure 1.**
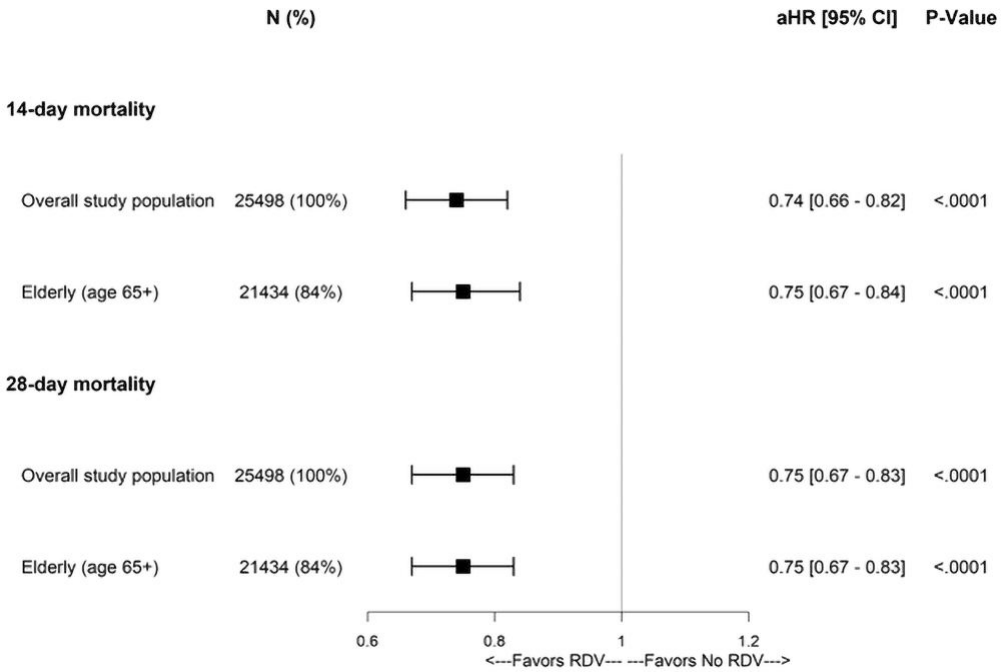
14- and 28-day mortality in patients hospitalized for COVID-19 in 1:1 propensity score (PS)–matched RDV and No RDV groups. Estimates were adjusted for baseline covariates such as age, sex, race, insurance type, comorbidity burden, indicators of disease severity upon admission. aHR indicates adjusted hazard ratio; CI, confidence interval, RDV, remdesivir-treated PS matched group; No RDV, remdesivir-untreated PS matched group.

Overall, the initiation of remdesivir in patients hospitalized for SARS-CoV-2 infection was associated with a reduction in mortality rate (6.2% versus 8.1% in the RDV and No RDV groups, respectively) in the period from admission to discharge. This corresponds to 242 lives saved and an estimated NNT of 52.7 to avoid one death. In the elderly subgroup, this clinical benefit was somewhat greater (a 6.9% versus 9.0% mortality rate in the RDV and No RDV groups, respectively), corresponding to 230 lives saved and an NNT of 46.6. These differences in in-hospital mortality were statistically significant in both the overall population and the elderly subgroup (*P* < 0.0001; Table 2). Furthermore, a significant reduction in mortality risk at 14 and 28 days was observed overall and among the elderly (adjusted HR for RDV versus No RDV [95% CI], 0.75 [0.67-0.83], *P* < 0.001 for both the overall population and the elderly), corresponding to a 25% decrease in relative risk of death during hospitalization (see Figure 1).

**Table 2.** Clinical and Economic Outcomes of Patients Hospitalized for SARS-CoV-2 infection

Population	No. of patients	Deaths		Mean (SD) cost	NNT (RDV versus No RDV)
		No. (%)	*P* value (RDV versus No RDV)		
**Overall**					
RDV	12,749	795 (6.2)	<0.0001	$18,329 ($18,479)	NA
No RDV	12,749	1,037 (8.1)		$14,845 ($17,296)	52.7
**Elderly (65+)**					
RDV	10,717	738 (6.9)	<0.0001	$18,142 ($17,118)	NA
No RDV	10,717	968 (9.0)		$14,772 ($15,879)	46.6

Abbreviations: NA, not applicable; No RDV, remdesivir-untreated patients; RDV, remdesivir-treated patients; SARS-CoV-2, severe acute respiratory syndrome coronavirus 2; SD, standard deviation.

Among patients requiring supplemental oxygen at baseline, remdesivir-treated patients exhibited a significantly lower mortality rate than those who did not receive remdesivir (8.1% versus 10.9%, respectively; *P* < 0.0001). This finding indicates that the mortality benefit of remdesivir persists in patients with greater disease severity and supports its effectiveness in subgroups commonly prioritized for antiviral therapy.

The mean length of stay was shorter among remdesivir-treated patients as compared to those who did not receive remdesivir (6.7 days versus 7.5 days, respectively), representing a reduction of 0.8 days per hospitalization. The mean total hospitalization cost was $18,329 in the RDV group and $14,845 in the No RDV group, corresponding to an estimated average cost per hospital day of approximately $2,735 and $1,980, respectively. Although overall hospitalization costs were higher among remdesivir-treated patients, the reduction in length of stay indicates more efficient use of hospital resources and improved patient throughput.

The average hospitalization cost in the RDV group was $18,329, which was $3,484 higher than the $14,845 average in the No RDV group, a modest difference given the clinical context.^4^ The majority of the difference was due to the cost of remdesivir, which ranges from $2,340 to $3,120 per treatment course, depending on insurance coverage.^23^

Detailed outcomes for nonelderly age subgroups, including mortality rates and costs, are provided in supplementary eTable 1.

The cost-effectiveness analysis demonstrated that remdesivir is a cost-effective treatment option for hospitalized patients with COVID-19. In the overall population, remdesivir treatment was associated with a cost of $20,419 per LYG and $24,003 per QALY gained. Among the elderly subgroup, the results were even more favorable ($19,150 per LYG and $22,529 per QALY gained), reflecting the greater absolute benefit in this higher-risk population (see Table 3).

**Table 3.** Cost-Effectiveness of Remdesivir (Overall Population and Elderly Subgroup)

Outcome	RDV group	No RDV group	Incremental difference
**Overall population**			
Mean hospitalization cost	$18,329	$14,854	$3,484
Total LYLs	7,678	9,854	2,175
Total QALYs lost	6,533	8,403	1,850
ICER (cost per LYL)	NA	NA	$20,419
ICER (cost per QALY, overall population)	NA	NA	$24,003
**Elderly subgroup**			
Mean hospitalization cost	$18,142	$14,772	$3,370
Total LYLs	6,052	7,938	1,886
Total QALYs lost	5,144	6,747	1,603
ICER (cost per LYL)	NA	NA	$19,150
ICER (cost per QALY, overall population)	NA	NA	$22,529

Abbreviations: No RDV, remdesivir-untreated patients; RDV, remdesivir-treated patients; ICER, incremental cost-effectiveness ratio; LYL, life-years lost; QALY, quality-adjusted life-year.

## Discussion

Initiation of remdesivir upon admission in patients hospitalized for SARS-CoV-2 infection was associated with significantly reduced mortality compared to not initiating remdesivir on admission (6.2% versus 8.1%, respectively; *P* < 0.0001), yielding a 1.9% absolute reduction in in-hospital mortality in the overall population. Importantly, this clinical benefit in both the overall population and in the elderly was achieved with only modest increases in costs, underscoring the value of considering drug costs in the context of clinical benefits realized (eg, lives saved) when hospitals plan for the treatment of patients hospitalized for SARS-CoV-2 infection.

It is important to highlight that the in-hospital mortality among patients with COVID-19 in the US has varied over different phases of the pandemic. Mortality rates as high as 28% were reported early in the pandemic, declining during periods of predominance of new strains of SARS-CoV-2 to 13.1% during the Omicron period.^17,24–26^ While the decline in mortality rates suggests improvement of SARS-CoV-2 management over time and likely includes the positive impact of widespread vaccination, the rates of SARS-CoV-2 infection continue to remain higher than those for other infectious diseases, as previously noted.^8^ To further illustrate this point, our calculated NNTs (52.7 overall and 46.6 in elderly subjects) are slightly lower than those reported for antiviral treatments in other infectious diseases such as influenza, although the latter are often reported in less severely ill outpatient populations. For example, the NNT to prevent one hospitalization for oseltamivir in high-risk patients with influenza has been estimated at 80 to 100.^27^ While cross-disease comparisons must be interpreted cautiously due to differences in setting and outcomes (eg, outpatient management of influenza versus inpatient COVID-19 care), this comparison clearly shows the ongoing value to hospitals of remdesivir and its effectiveness in SARS-CoV-2 management despite possible reductions in overall mortality risk arising from SARS-CoV-2 infection during the transition from pandemic to endemic phase.

From an economic perspective, prior cost-effectiveness evaluations from NICE^17,26^ and US-ICER^18^ have assessed remdesivir’s cost-effectiveness in treating SARS-CoV-2 infection, emphasizing its effectiveness in reducing mortality, particularly for patients with higher baseline risk. Despite NICE’s commonly used willingness-to-pay (WTP) threshold of £30,000 (∼$38,000 in the US) per QALY, remdesivir was considered cost-effective when the untreated mortality risk exceeded a certain threshold, particularly in the double-digit range. Specifically, the NICE appraisal identified the threshold underlying mortality risk as approximately 14% at 28 days, below which remdesivir’s incremental cost-effectiveness ratio exceeded the £30,000 per QALY threshold and thus the drug was no longer cost-effective.^17^ However, the NICE evaluation relied on older mortality data that may not fully reflect more recent findings. A broader synthesis of data, including updated findings from the Solidarity trial,^28^ showed that remdesivir provided a statistically significant reduction in mortality risk (relative risk, 0.87 [95% CI, 0.76-0.99]; *P* = 0.03), a result that suggests that the original conclusion of NICE related to the cost-effectiveness of remdesivir remains viable even during the transition of SARS-CoV-2 from pandemic to endemic status.

Consistent with prior trial findings, our subgroup analysis of patients requiring supplemental oxygen at baseline showed that remdesivir-treated patients had a significantly lower in-hospital mortality than those not receiving remdesivir (8.1% versus 10.9%, respectively; *P* < 0.0001). This suggests that the mortality benefit of remdesivir extends to patients with greater disease severity, reinforcing its role in treatment pathways that prioritize higher-risk hospitalized individuals.

Our analysis showed that remdesivir is a cost-effective treatment in hospitalized patients with COVID-19, with values falling well below the commonly accepted US WTP threshold of $100,000 per QALY. Evidence from prior studies^18,26,29^ shows that remdesivir remains cost-effective in real-world scenarios even as baseline mortality rates have declined over time, as previously noted.

Our findings indicate that the observed reductions in mortality and favorable cost-effectiveness profile associated with remdesivir are primarily driven by patients 65 years of age or older, reflecting the higher baseline risk and greater absolute benefit in this population. This age-stratified effect highlights the subgroup in which remdesivir provides the most meaningful clinical and economic value. From a hospital perspective, these findings support prioritizing remdesivir use among elderly and other high-risk hospitalized patients, where the impact on outcomes and resource utilization is most pronounced.

The consistently greater absolute benefit observed in patients age 65 or older indicates that this subgroup derives the most meaningful clinical and economic value from remdesivir treatment. These findings support prioritizing remdesivir use among elderly hospitalized patients, where reductions in mortality and favorable cost-effectiveness outcomes are most pronounced and most relevant for hospital decision-making.

Collectively, these findings highlight the clinical and economic benefit of remdesivir, particularly for high-risk populations such as the elderly. The potential for nonseasonal surges in COVID-19 cases, with peaks occurring outside the typical winter respiratory virus season, presents an ongoing challenge for hospital resource planning. Unlike seasonal influenza,^10,25^ these unpredictable waves can strain hospital capacity, requiring hospitals to remain adaptable in their budgeting strategies. Given the dynamic nature of COVID-19 epidemiology, healthcare systems must proactively assess treatment availability and financial considerations to maintain preparedness for future surges. In this context, remdesivir is a clinically effective and economically sound investment for hospitals, a critical value proposition given the current US healthcare funding landscape. Its proven ability to save lives, combined with the minimal investment required to realize the lives saved, offers a clear value proposition as a treatment option for hospitalized patients that is further supported by the consistent effectiveness of remdesivir across SARS-CoV-2 viral variants^2,30^ and its role in reducing mortality.^11,12^

Given the highly variable and unpredictable pattern of COVID-19 surges, hospital pharmacies face ongoing difficulty in maintaining optimal remdesivir inventory levels, with the potential for excess supply during periods of low disease activity and insufficient availability during peak demand. This operational uncertainty highlights the broader challenges hospitals encounter in managing antiviral resources in a dynamic and evolving care environment. By quantifying the magnitude of mortality reduction, the numbers needed to treat, the modest incremental hospitalization costs, and the reduction in length of stay associated with remdesivir, this analysis offers concrete parameters that hospitals can use to inform evidence-based forecasting, formulary planning, and inventory management decisions for remdesivir in anticipation of future surges.

As a rigorous assessment of the clinical benefits of remdesivir treatment and the associated economic value requires a comparison using matching of treated and untreated populations, the primary strength of this study arises from its use of PS matching to meet this end. To improve the accuracy of our cost estimates, we excluded records where hospital costs were reported as zero but had not been formally verified. These cases likely reflected billing errors or temporary government coverage rather than true hospital expenses. Removing these records helped prevent underestimating the actual costs borne by the hospitals. Finally, the PHD is one of the few sources that provides granular data on actual costs (as opposed to hospital charges) at both the hospital and departmental levels for a diverse population of hospitalized patients across the entire US, enabling a comprehensive evaluation of the true economic benefit of remdesivir treatment that encompasses a broad range of clinical care settings and insurance types.

From a methodological perspective, this analysis employed real-world hospitalization costs and mortality outcomes from a large, nationally representative dataset to generate directly observed cost-effectiveness estimates. This approach is consistent with previously published real-world evidence–based cost-effectiveness studies (eg, Chiou SS et al, 2024; Kepka S et al, 2023; Serra-Burriel M et al, 2023)^31–33^ that used administrative claims or hospital data to assess value. This analysis uses observed data to estimate both cost and effectiveness outcomes. As such, the cost-effectiveness estimates derived in this study reflect a pragmatic, real-world estimate of value and are representative of outcomes in routine clinical practice rather than the more typical approach of extrapolation and modeling based on trial-based efficacy. They thereby offer relevant and representative evidence for health systems managing COVID-19 resource allocation and supporting decision-making based on outcomes seen in routine care rather than extrapolated trial-based efficacy.

However, there are limitations to this study. First, the retrospective design limits the ability to establish causal relationships related to clinical outcomes. While this introduces some uncertainty, it does not necessarily weaken the findings, as the large, real-world dataset still provides valuable insights into the effectiveness and cost of remdesivir. Second, unmeasured confounders may introduce bias, potentially skewing the results either toward overestimating or underestimating the true effect of remdesivir. Overall, these limitations do not substantially diminish the overall validity of the conclusions, particularly given the consistency of the results with prior studies. Given the retrospective nature of this real-world analysis and the potential for residual confounding, the observed benefits of remdesivir should be interpreted with appropriate caution, with the most consistent and clinically meaningful impact observed among higher-risk subgroups, particularly elderly hospitalized patients.

In addition, the cost-effectiveness analysis did not incorporate a state-transition model or health state–specific utilities. Instead, we applied a static utility weight based on general population norms, which may not fully reflect changes in quality of life during or after hospitalization. Furthermore, we did not model time-dependent transition probabilities for survival or recovery, limiting the ability to capture long-term dynamic outcomes. While our approach increases transparency and applicability in the real-world setting and for time frames most relevant to hospitals, the nuances of the approach highlighted should be considered carefully before contextualizing the quantitative results amongst other model-based cost-effectiveness or cost-utility analysis results.

## Conclusion

Our findings underscore the value of initiating remdesivir in hospitalized patients for SARS-CoV-2 infection, where the treatment was associated with reduced in-hospital mortality at modest incremental cost. These results, generated from a large real-world dataset and subject to the inherent limitations of observational research, suggest that remdesivir can be an economically reasonable component of hospital management strategies for higher-risk patients rather than a uniformly high-impact intervention across all hospitalized individuals. With the US government no longer funding COVID-19 efforts, hospitals must consider both the clinical benefits and the economic implications of remdesivir, especially in these high-risk groups, when making budgeting and formulary decisions. Our results align with previous evaluations from NICE and US-ICER and recent pandemic-era trials,^12,28^ highlighting remdesivir’s role as an economically sustainable intervention and supporting its continued place in SARS-CoV-2 infection protocols and hospital order sets.

## Supplementary Material

zxag038_Supplementary_Data

## Data Availability

The data supporting this study’s findings are available from Premier, Inc. (https://www.premierinc.com/). Restrictions apply to the availability of these data, which were used under license for this study.

## Additional information

The authors provided the following information regarding their contributions to this article: All authors contributed substantially to the manuscript and agreed to the final submitted version.
