# Supplementary material for: Management of patients hospitalized for SARS-CoV-2 infection: A real-world economic evaluation from the hospital perspective
Source: Am J Health Syst Pharm. 2026 Feb 11;83(Suppl 3):S2952–61. doi: 10.1093/ajhp/zxag038 (PMC13070688; doi:10.1093/ajhp/zxag038)
Supplement: zxag038_Supplementary_Data [file zxag038_supplementary_data.docx]

## **eTable 1.** Clinical and Economic Outcomes in the Hospitalized SARS-CoV-2 Population among those <65 years of age

| **Age Group** | **Treatment Group** | **Number of Patients** | **Deaths**  **______________________** | | **Mean Cost ($)** |
| --- | --- | --- | --- | --- | --- |
|  |  |  | **n (%)** | **P-value (RDV vs. No RDV)** |  |
| 18-49 | RDV | 441 | 7 (1.6) | 0.7799 | 19,724 |
|  | No RDV | 441 | 6 (1.4) |  | 16,103 |
| 50-64 | RDV | 1,591 | 50 (3.1) | 0.2130 | 19,202 |
|  | No RDV | 1,591 | 63 (4.0) |  | 14,991 |

Abbreviations: N, number of patients; No RDV, remdesivir untreated patients; RDV, remdesivir treated patients.
